# Lifetime impact of being underweight or overweight/obese during childhood in Vietnam

**DOI:** 10.1186/s12889-022-13061-8

**Published:** 2022-04-04

**Authors:** Yeji Baek, Alice J. Owen, Jane Fisher, Thach Tran, Zanfina Ademi

**Affiliations:** 1 Present Address: School of Public Health and Preventive Medicine, Monash University, Melbourne, VIC Australia; 2 Research and Training Centre for Community Development, Hanoi, Vietnam; 3 Centre for Medicine Use and Safety, Faculty of Pharmacy and Pharmaceutical Sciences, Monash University, Melbourne, Australia

**Keywords:** Child, Underweight, Overweight, Malnutrition, Life table, Markov model, Vietnam

## Abstract

**Background:**

There is limited evidence about lifetime burden of child malnutrition. This study aimed to estimate the lifetime impact of being underweight or overweight/obese during childhood in Vietnam.

**Methods:**

We developed a life table model in combination with a Markov model for Vietnamese children aged 5–19 years and simulated until they reached 75 years of age or died using published data. The starting year was 2019 and the model estimated number of deaths, years of life lived and quality-adjusted life years (QALY) with an annual discount rate of 3%. We performed scenario, one-way, and probabilistic sensitivity analyses to assess the impact of uncertainties in input parameters.

**Results:**

The model estimated 9.68 million deaths (6.44 million men and 3.24 million women), 622 million years of life lived (317 million men and 305 million women), and 601 million QALYs (308 million men and 293 million women). Scenario analyses showed that the reduction in either underweight or overweight/obesity alone, and reduction in both underweight and overweight/obesity resulted in fewer deaths, more years of life lived and more QALYs gained. In the scenario where everyone was a healthy weight, the model estimated 577,267 fewer deaths (6.0% less), 2 million more years of life lived (0.3% more), and 3 million QALYs gained (0.6% more) over base-case results which represents current situation in Vietnam.

**Conclusions:**

Our results suggest that addressing underweight and overweight/obesity will contribute to reducing deaths and increasing years of life lived and QALYs. Policies and interventions in alignment with Sustainable Development Goals to address underweight and overweight/obesity are necessary to achieve health for all.

**Supplementary Information:**

The online version contains supplementary material available at 10.1186/s12889-022-13061-8.

## Background

Optimal health and nutrition during childhood and adolescence underpin personal, national and international development. Malnutrition during childhood and adolescence increases the risk of morbidity and mortality, impairs cognitive development and reduces work productivity in later life [1–3]. In particular, improving nutrition among children and adolescents 5 to 19 years is crucial as it affects the timing and pattern of puberty, adult height, muscle, as well as risk of non-communicable diseases in later life. It is critical periods to ensure a healthy transition to adulthood [1, 4].

While undernutrition problems still remain as a major public health concern in low-and middle-income countries, overweight and obesity have increased globally [5]. The double burden of malnutrition, which is the coexistence of undernutrition and overweight/obesity, has increased in the poorest low-and middle-income countries [6]. In particular, overweight and obesity have increased, mainly due to rapid changes in the food system including the availability of cheap ultra-processed food and beverages, and major reductions in physical activity [6]. Actions to simultaneously prevent or reduce the risk of nutritional deficiencies and obesity have been proposed to address malnutrition in a more holistic way [7].

In Vietnam, rapid economic development and urbanization have also shifted the health and nutritional status of the population. The National Nutrition Strategy for Vietnam envisions reduced child malnutrition including both stunting and overweight/obesity [8], however, studies have shown that double burden of malnutrition in children still exists [9, 10]. The General Nutrition Survey in Vietnam discovered that the prevalence of stunting has decreased from 23.4% in 2010 to 14.8% in 2020 but the prevalence of overweight and obesity has increased from 8.5% to 19% over the same period among children aged 5–19 years [11]. However, there is limited evidence about the long-term impact of this changing picture of child and adolescent malnutrition, which is necessary for policy makers to understand the magnitude of burden, formulate policy and design intervention programs. When lifetime data are not available, modeling can be a useful way to quantify and structure the decision problem to maximize population health within available resources [12, 13]. A few modeling studies have estimated impacts of childhood overweight and obesity from Germany, the United States and Thailand, highlighting long-term burden regarding costs and quality-adjusted life years (QALY) [14–17]. However, the lifetime burden of undernutrition such as stunting, wasting or being underweight has not been included, and the long-term impact of child malnutrition in Vietnam is unknown. Therefore, this study aimed to estimate the lifetime burden of being underweight or overweight/obese during childhood in terms of deaths, years of life lived and QALY at the population level in Vietnam.

## Methods

### Model description

**Fig. 1:**
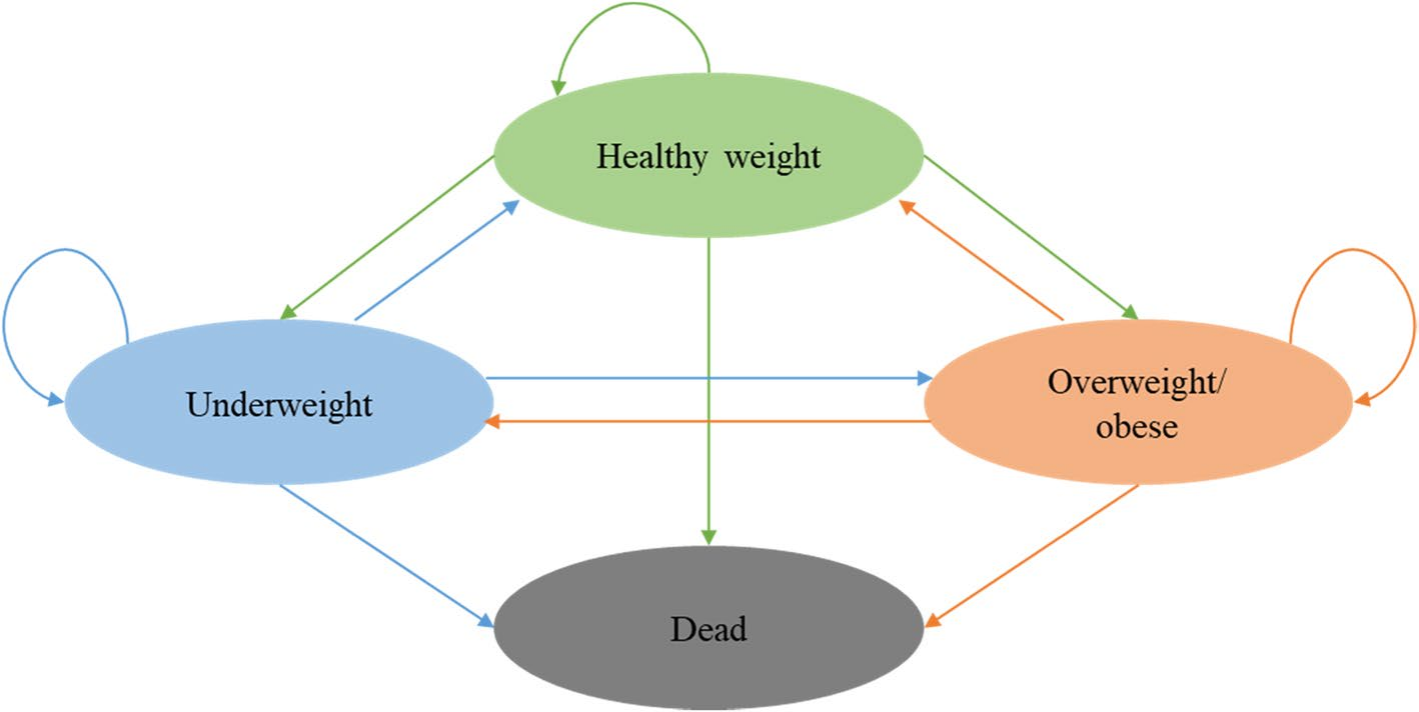
Model description including four health states

We developed a state-of-the-art life table model in combination with a Markov model with four health states, including underweight, healthy weight, overweight/obesity and death (Fig. 1). The Markov model is a commonly used analytical framework where individuals move between health states over time, and costs and health outcomes are aggregated accordingly for a modelled cohort [18]. We developed age and sex-specific life tables for Vietnamese children aged 5–19 years and simulated until they reached 75 years of age or died. The life expectancy at birth in Vietnam was 75 years in 2019 according to the World Bank [19]. The modeled population was classified into underweight, healthy weight, overweight/obese weight statuses at the starting point of the model, and then transitioned between weight status categories until 75 years of age or death. The model estimated number of deaths, years of life lived and QALYs with an annual discount rate of 3%. The discount rate applied was derived from the WHO Guide to Cost-effectiveness Analysis [20] and recent cost-effectiveness studies in Vietnam [21, 22] to account for outcomes that occur in the future.

### Data sources

#### Demographic profile and mortality

The model inputs with data sources are shown in Table 1 and all values of each parameter are listed in Additional file 1. The demographics of the model population by sex and 5-year age groups were from the Vietnam General Statistics Office [23]. We derived the all-cause mortality rates by sex and 5-year age group from the life table for Vietnam published by the WHO Global Health Observatory data repository 2019 (Additional file 1: Table 1-C) [24]. The mortality rates by single age were extrapolated using exponential functions as a best fit.

**Table 1:** Model inputs and data sources

Input	Description	Data source	Values
Population	Vietnamese children aged 5–19 years	2019 Viet Nam Population and Housing Census [23]	See Table 2
Health states	Prevalence of underweight and overweight/obesity by sex and age	NCD Risk Factor Collaboration 2016 [25, 26]	See Additional file 1: Table 1-A
Transition probability between health states	Transition probability between weight status by sex and age (i.e., underweight to underweight, underweight to healthy weight or underweight to overweight/obesity)	5–22 years old: Young Lives Study [27] 23 years and over: China Health and Nutrition Survey [28]	See Additional file 1: Table 1-B
Mortality	All-cause mortality rates by sex and age	WHO life table for Vietnam from Global Health Observatory data repository 2019 [24]	See Additional file 1: Table 1-C
	Mortality risk by weight status for those 20 years and over	The Global BMI Mortality Collaboration [29]	See Additional file 1: Table 1-D
Quality-adjusted life-years	Quality-adjusted life-years by sex, age and weight status	Mai VQ et al. Reference Data and Known-Groups Validity of the EQ-5D-5L for Vietnam [30]	See Additional file 1: Table 1-E

The mortality risk for underweight and overweight/obesity groups in adults were derived from the contemporary meta-analysis by the Global BMI Mortality Collaboration [29]. We applied differential mortality risks for adults by weight status categories using data from East Asia, as Vietnam-specific data were not available (Additional file 1: Table 1-D). Due to lack of data, we were not able to apply differential mortality risks for underweight and overweight/obesity groups in children. Therefore, our assumptions for those under the age of 20 are conservative.

#### Weight status and transition probabilities

The model consists of underweight, healthy weight, and overweight/obesity based on the WHO reference [31, 32]. The prevalence of underweight, healthy weight and overweight/obesity in Vietnam by sex and age were from the NCD Risk Factor Collaboration (Additional file 1: Table 1-A) [25, 26]. Underweight is defined as a BMI-for age below -2 Z-scores and overweight/obesity is defined as a BMI-for age above 1 Z-scores for children aged 5—19 years. In adults, underweight is defined as a BMI less than 18.5 kg/m^2^ and overweight/obesity is defined as a BMI greater than or equal to 25 kg/m^2^.

We used two cohort data sources to estimate the transition probabilities between different weight status (Additional file 1: Table 1-B). For those aged 5–22 years, we used Vietnam data from the Young Lives study [27] and for adults aged 23 years and over, we used data from the China Health and Nutrition Survey [28] as Vietnam-specific data were not available for adults. Briefly, Young Lives is an international longitudinal study which aims to investigate the changing nature of childhood poverty in four low-and-middle-income countries, including Vietnam, Ethiopia, India and Peru with around 3,000 participants in each country [27]. The China Health and Nutrition Survey is an ongoing open cohort to cover key public health risk factors and health outcomes, demographic, social and economic factors with around 18,764 participants [28]. Survey descriptions can be found elsewhere [27, 28].

We combined overweight and obese categories given that the prevalence of obesity was less than 3% in the Young Lives study. Estimating the transition probabilities was not possible due to the small sample size in the obesity group in the Young Lives study [27]. For instance, the sample size to estimate the change in weight status within the obese group was only 2 boys and 1 girl aged 15 years. Combining overweight and obesity has been applied to another study which also used the data from the Young Lives study to model trajectories in stunting and overweight status in children in Ethiopia, India, Peru and Vietnam [33]. In a modelling study of Nigerian women, malnutrition was classified into underweight (severe thinness and undernourished) and overweight (overweight and obesity) [34].

### Quality of adjusted life years

Quality of life data were derived from a Vietnamese study [30]. For those aged 18–34 years, the utility scores were estimated from general population of 562 adults in Vietnam using the EQ-5D-5L instrument, but due to unavailability of weight data, the same utility values regardless of weight status were applied by sex and age groups. In the same Vietnamese study [30], weight data were available for different population in Ho Chi Minh City (*n* = 1,296), so utility values by sex, age groups and weight status for those aged 35 years and over were estimated accordingly. We applied utility values of 1 for children aged 5–17 years as utility data for children were not available. Utility values are presented in Additional file 1: Table 1-E. Utility scores as described above were then multiplied with years of life lived to generate QALYs.

### Scenario analyses

Several scenario analyses were conducted to demonstrate the impact of being underweight or overweight/obese under various scenarios. The scenarios included a reduction in the prevalence of underweight and overweight/obesity, and changes in transition probabilities to underweight or overweight/obesity by 10%, 20% and 50%. Scenario analyses also estimated the outcomes from having no underweight and overweight/obesity in the population (i.e., an entirely healthy weight population) to estimate the total impact of malnutrition. To reflect the increasing trends in overweight/obesity, the model simulated a 10% increase in overweight/obesity. We also varied discount rates as 0%, 4% and 5%.

### Sensitivity analyses

We performed one-way and probabilistic sensitivity analyses to assess the impact of uncertainties in input parameters on outcomes. As for one-way sensitivity analyses, we varied values of prevalence of underweight and overweight/obesity, transition probabilities between weight status and QALYs one by one based on lower and upper 95% confidence intervals (CI). We conducted probabilistic sensitivity analysis through second order Monte Carlo simulations by varying values of prevalence of underweight and overweight/obesity, transition probabilities between weight status, mortality risks, and utilities simultaneously. We ran the Monte Carlo simulation for 10,000 iterations through random sampling from values represented by different distributions to consider uncertainty in the model inputs. We assumed a uniform distribution for prevalence and transition probabilities, a lognormal distribution for mortality risks, and a beta distribution for utility scores as indicated in Additional file 1.

We used Microsoft Excel 2016 and @Risk 8.2 to construct the model and conduct scenario and sensitivity analyses. The model is validated through face validity and internal validity to reduce errors and better represent reality.

### Ethical considerations

Ethics approval was not required as this study used publicly available data to construct the model. Regarding secondary data used in the study, ethics approvals for the Young Lives study were obtained in each study country and by the Social Sciences and Humanities Inter-Divisional Research Ethics Committee at the University of Oxford [27, 35]. The China Health and Nutrition Survey was approved by institutional review boards at the University of North Carolina at Chapel Hill and the National Institute of Nutrition and Food Safety [36]. The quality of life study in Vietnam was approved by the Ethical Review Board for Biomedical Research at the Hanoi University of Public Health [30]. All methods were carried out in accordance with relevant guidelines and regulations, and the study participants or caregivers of minors provided their informed consent [27, 30, 35, 36].

## Results

The model simulated 22,058,773 Vietnamese children aged 5–19 years (11,444,303 boys and 10,614,470 girls) as presented in Table 2. The prevalence of underweight ranged from 12% to 18% in boys and 6% to 19% in girls depending on ages. The prevalence of overweight/obesity ranged from 7% to 22% in boys and 6% to 11% in girls depending on ages. There were more girls with healthy weight than boys across all the age groups.

**Table 2:** Modelled population

	Population		Underweight		Healthy weight		Overweight/obesity	
Age	Boys	Girls	Boys	Girls	Boys	Girls	Boys	Girls
5	4,354,887	3,977,832	12.3%	12.9%	66.9%	76.3%	20.8%	10.9%
6			13.1%	12.7%	66.3%	76.2%	20.6%	11.0%
7			14.1%	14.2%	64.8%	74.6%	21.1%	11.2%
8			15.2%	16.4%	63.2%	72.4%	21.7%	11.2%
9			16.2%	18.5%	62.1%	70.5%	21.7%	11.0%
10	3,737,030	3,482,807	17.0%	19.4%	62.2%	70.1%	20.8%	10.5%
11			17.6%	18.9%	63.6%	71.4%	18.8%	9.8%
12			17.7%	17.1%	65.9%	73.9%	16.4%	9.0%
13			17.6%	14.8%	68.5%	77.0%	13.9%	8.2%
14			17.1%	12.2%	71.3%	80.3%	11.6%	7.5%
15	3,352,386	3,153,831	16.3%	9.9%	73.9%	83.2%	9.7%	6.9%
16			15.4%	8.0%	76.3%	85.5%	8.3%	6.5%
17			14.3%	6.8%	78.4%	86.8%	7.3%	6.4%
18			13.2%	6.3%	79.9%	87.1%	6.9%	6.5%
19			14.5%	8.2%	76.5%	85.1%	8.9%	6.8%

### Deaths, years of life lived, quality adjusted life years

As a base-case with current prevalence of underweight and overweight/obesity and transition probabilities between weight status for Vietnamese children aged 5–19 years simulated until they reached 75 years of age, the model estimated 9.68 million deaths (6.44 million men and 3.24 million women), 622 million years of life lived (317 million men and 305 million women), and 601 million QALYs (308 million men and 293 million women) (Table 3). There were more deaths, years of life lived and QALYs among healthy weight groups than underweight or overweight/obese groups reflecting the greater size of the healthy weight group population.

**Table 3:** Deaths, years of life lived and QALYs followed up until 75 years of age (base-case)

	Deaths				Years of life lived				QALYs			
Age group	UW	HW	OWB	Total	UW	HW	OWB	Total	UW	HW	OWB	Total
Men												
5–9	167,587	1,293,769	991,414	2,452,770	10,593,642	79,192,463	32,748,453	122,534,557	10,434,860	77,462,671	31,834,473	119,732,004
10–14	143,245	1,107,671	851,036	2,101,952	7,834,098	64,933,117	28,886,431	101,653,646	7,675,863	63,209,353	27,975,622	98,860,838
15–19	127,531	990,103	764,197	1,881,831	5,629,735	57,314,762	29,426,907	92,371,403	5,455,401	55,414,529	28,425,547	89,295,477
Total	438,363	3,391,543	2,606,647	6,436,552	24,057,475	201,440,341	91,061,791	316,559,607	23,566,124	196,086,554	88,235,642	307,888,320
Women												
5–9	88,928	595,359	533,459	1,217,746	13,170,646	78,943,176	23,559,900	115,673,722	12,777,945	76,838,070	22,233,450	111,849,465
10–14	77,645	519,870	467,152	1,064,667	10,938,964	65,405,842	22,087,427	98,432,233	10,540,083	63,267,813	20,740,223	94,548,119
15–19	69,864	468,856	423,274	961,994	9,496,629	57,810,961	23,556,472	90,864,062	9,069,451	55,428,540	22,053,054	86,551,045
Total	236,437	1,584,085	1,423,885	3,244,407	33,606,239	202,159,980	69,203,799	304,970,018	32,387,479	195,534,424	65,026,726	292,948,629
Men and women												
5–9	256,515	1,889,128	1,524,873	3,670,515	23,764,287	158,135,639	56,308,353	238,208,280	23,212,805	154,300,741	54,067,923	231,581,469
10–14	220,890	1,627,541	1,318,188	3,166,619	18,773,062	130,338,959	50,973,858	200,085,880	18,215,946	126,477,167	48,715,844	193,408,957
15–19	197,395	1,458,959	1,187,471	2,843,825	15,126,364	115,125,723	52,983,378	183,235,466	14,524,852	110,843,070	50,478,601	175,846,523
Total	674,800	4,975,627	4,030,532	9,680,959	57,663,714	403,600,321	160,265,590	621,529,625	55,953,603	391,620,978	153,262,368	600,836,948

*UW* Underweight, *HW* Healthy weight, *OWB* Overweight/obesity, *QALYs* Quality-adjusted life years

### Scenario analyses

Scenario analyses demonstrated the changes in impact of being underweight or overweight/obese. Study outcomes under various scenarios are presented in Table 4 and the percentage difference compared to the base-case results are summarized in Additional file 2. The reduction in either underweight or overweight/obesity alone, and reduction in both underweight and overweight/obesity resulted in fewer deaths, more years of life lived and more QALYs gained. Reducing both underweight and overweight/obesity had a greatest impact on outcomes followed by reducing overweight/obesity alone and underweight alone. The total number of deaths decreased to 9.6 million, 9.5 million, 9.3 million when the prevalence of underweight and overweight/obesity and transition probabilities to underweight and overweight/obesity were reduced by 10%, 20% and 50%, respectively compared to the base-case results (9.7 million). In the scenario where everyone was a healthy weight, the model estimated 9.1 million deaths, which is 577,267 fewer deaths or 6.0% less (5.4% men and 7.0% women), 624 million years of life lived, which is 2 million more years of life lived or 0.3% more (0.5% men and 0.2% women), and 604 million QALYs, which is 3 million more QALYs gained or 0.6% more (0.4% men and 0.7% women) than base-case results. A 10% increase in overweight/obesity estimated 116,654 more deaths (1.2%), 494,474 fewer years of life lived (0.08%), and 897,866 fewer QALYs gained (0.15%) than base-case results. Compared to the base-case results with an annual discount rate of 3%, the model estimated 181 million fewer years of life lived and 174 million fewer QALYs with an annual discount rate of 5%.

**Table 4:** Scenario analyses

	Deaths			Years of life lived			QALYs		
	Men	Women	Total	Men	Women	Total	Men	Women	Total
Base-case	6,436,552	3,244,407	9,680,959	316,559,607	304,970,018	621,529,625	307,888,320	292,948,629	600,836,948
Underweight									
10% reduction	6,421,736	3,235,584	9,657,321	316,606,814	304,996,333	621,603,146	307,931,137	292,973,262	600,904,399
20% reduction	6,409,303	3,228,321	9,637,623	316,647,304	305,018,748	621,666,052	307,967,970	292,994,449	600,962,419
50% reduction	6,381,666	3,212,633	9,594,299	316,740,400	305,069,616	621,810,016	308,053,003	293,043,225	601,096,229
No underweight	6,353,624	3,197,351	9,550,975	316,839,661	305,122,583	621,962,244	308,144,159	293,095,050	601,239,209
Overweight/obesity									
10% reduction	6,388,001	3,212,810	9,600,811	316,799,775	305,066,553	621,866,328	308,107,086	293,350,407	601,457,493
20% reduction	6,352,770	3,189,463	9,542,233	316,972,376	305,137,893	622,110,269	308,264,921	293,646,644	601,911,565
50% reduction	6,287,936	3,145,675	9,433,610	317,285,097	305,271,666	622,556,762	308,552,780	294,198,808	602,751,588
No overweight/obesity	6,235,980	3,109,914	9,345,895	317,530,092	305,380,910	622,911,002	308,780,579	294,644,694	603,425,273
10% increase	6,508,022	3,289,591	9,797,613	316,203,016	304,832,135	621,035,151	307,564,572	292,374,511	599,939,082
Underweight and overweight/obesity									
10% reduction	6,370,643	3,202,308	9,572,951	316,854,652	305,096,279	621,950,931	308,157,110	293,380,775	601,537,885
20% reduction	6,317,247	3,167,877	9,485,124	317,085,142	305,197,887	622,283,029	308,368,234	293,711,920	602,080,154
50% reduction	6,201,845	3,092,983	9,294,828	317,561,263	305,414,978	622,976,241	308,808,152	294,372,223	603,180,376
All healthy weight									
	6,085,928	3,017,764	9,103,692	318,017,145	305,630,002	623,647,147	309,234,187	294,969,934	604,204,121
Discount rate									
0%	6,436,552	3,244,407	9,680,959	642,878,246	642,016,057	1,284,894,303	622,587,009	611,044,424	1,233,631,433
4%	6,436,552	3,244,407	9,680,959	264,405,312	252,724,245	517,129,557	257,500,197	243,390,933	500,891,130
5%	6,436,552	3,244,407	9,680,959	225,814,801	214,508,933	440,323,734	220,187,086	207,063,618	427,250,704

*QALYs* Quality-adjusted life years

### Sensitivity analyses

One-way sensitivity analyses showed that transition probabilities of being or staying underweight or overweight/obese have a greater impact on years of life lived and QALYs than the prevalence of underweight and overweight/obesity (Table 5). Using the values of lower CI of prevalence of underweight and overweight/obesity or transition probabilities of being/staying underweight and overweight/obesity resulted in fewer deaths, more years of life lived, and more QALYs than base-case results.

**Table 5:** One-way sensitivity analyses

	Deaths			Years of life lived			QALYs		
	Men	Women	Total	Men	Women	Total	Men	Women	Total
Prevalence of underweight and overweight/obesity									
Lower confidence interval	6,436,550	3,244,407	9,680,957	316,559,712	304,970,027	621,529,738	307,888,420	292,948,637	600,837,057
Upper confidence interval	6,436,555	3,244,407	9,680,963	316,559,456	304,970,012	621,529,468	307,888,175	292,948,623	600,836,798
Transition probability of being/staying underweight, overweight/obesity									
Lower confidence interval	6,352,656	3,196,077	9,548,733	316,900,839	305,109,141	622,009,980	308,212,395	293,275,208	601,487,603
Upper confidence interval	6,592,227	3,328,217	9,920,444	315,848,262	304,708,614	620,556,876	307,208,899	292,419,174	599,628,073
QALYs									
Lower confidence interval	6,436,552	3,244,407	9,680,959	316,559,607	304,970,018	621,529,625	305,044,247	289,241,789	594,286,036
Upper confidence interval	6,436,552	3,244,407	9,680,959	316,559,607	304,970,018	621,529,625	310,670,996	296,533,782	607,204,777

*QALYs* Quality-adjusted life years

Probabilistic sensitivity analyses estimated the mean and 95% CI for deaths, years of life lived and QALYs by sex (Table 6). In total, it estimated 9.7 million deaths (95% CI 9 to 10 million), 621 million years of life lived (95% CI 620 to 623 million) and 600 million QALYs (95% CI 571 to 617 million).

**Table 6:** Probabilistic sensitivity analyses

	Deaths			Years of life lived			QALYs		
	Mean		95% CI	Mean		95% CI	Mean		95% CI
Men	6,459,944	6,241,947	6,732,125	316,432,036	315,134,853	317,398,661	307,709,514	286,710,511	316,063,270
Women	3,257,661	3,120,190	3,425,340	304,925,611	304,398,387	305,346,475	292,782,212	269,206,310	303,572,918
Total	9,717,606	9,363,104	10,157,583	621,357,647	619,535,507	622,742,793	600,491,726	570,866,049	616,958,659

*QALYs* Quality-adjusted life years, *CI* confidence intervals

## Discussion

This study shows the lifetime impact of being underweight or overweight/obese for children in Vietnam in 2019. The model estimated 9.68 million deaths, 622 million years of life lived, and 601 million QALYs under the current prevalence of underweight and overweight/obesity among children who were followed up to age 75 years. The model also provided various estimations to quantify the impact of child underweight and overweight/obesity under different scenarios. Compared to the hypothetical scenario with an entirely healthy weight population, modeling of the current child weight status in Vietnam estimated the 577,267 excess deaths, 2 million fewer years of life lived, and 3 million fewer QALYs due to being underweight or overweight/obesity. The findings highlight the preventable losses and opportunity for possible gains from tackling underweight and overweight/obesity at the population level in Vietnam.

There is limited evidence on long-term impact of being underweight or overweight/obese especially in low-and middle-income countries, including Vietnam. Our study highlights the adverse consequences of underweight or overweight/obesity through several scenario analyses in Vietnam. The number of deaths would decrease by 1.3% (1.3% men and 1.5% women), 3.5% (3.1% men and 4.1% women) and 6.0% (5.4% men and 7.0% women) under the scenarios where no one was underweight, no one was overweight/obese and everyone was healthy weight, respectively. Our results are not directly comparable with previous studies from other countries due to differences in population, health systems and socioeconomic factors in their specific contexts. Still, a few studies pointed to the adverse health and economic consequences of being underweight or overweight/obese. A review paper from Brazil on long-lasting effects of undernutrition reported consistent findings, including a higher risk of diabetes in adulthood, hypertension, dyslipidemia and a lowered working capacity of manual workers, among other physiological impairments [37]. Similarly, a meta-analysis found that the hazard ratio for all-cause mortality was 1.51 for underweight (BMI 15.0 to 18.5), 1.07 to 1.20 for overweight (BMI 25.0 to 30.0) and 1.45 to 2.76 for obesity (BMI 30 to 60) in Asia, Australia and New Zealand, Europe, and North America [29]. In addition, a modelling study from Georgia showed that a 1%-point reduction in both overweight and obese adolescents could reduce lifetime medical care costs by $586 million and increase lifetime QALYs by 47,138 [17]. In Germany, the excess lifetime direct cost per person of inpatient and outpatient treatment cost due to overweight and obesity during childhood was €4,262 for men and €7,028 for women [14], and the excess lifetime indirect costs per person such as opportunity cost of lost productivity was €4,209 for men and €2,445 for women [15]. The use of an estimate of US $19,000 as the incremental lifetime medical cost of an obese child relative to a normal weight child was recommended in the United States [38]. Furthermore, another modeling study from the United States found that lifetime medical care costs would decrease by US$586 million and lifetime QALYs would increase by 47,138 with a 1%-point reduction in both overweight and obese adolescents [17]. These studies all highlighted adverse effects of being underweight or overweight/obese during childhood.

Reduction in deaths and increases in years of life lived and QALYs were greatest when reducing both underweight and overweight/obesity followed by reducing overweight/obesity alone and underweight alone. Vietnam is undergoing changes in health and nutrition outcomes due to economic development and urbanization. The increasing prevalence of nutrition-related chronic disease and westernization of the traditional Vietnamese diet have been reported [39, 40]. Given that undernutrition and food insecurity still exist in Vietnam with climate change risks [41–43], more emphasis on solving the double burden of malnutrition is required. Double-duty actions which aim to tackle both undernutrition and problems of overweight/obesity simultaneously have been proposed, based on the rationale that all forms of malnutrition share common drivers, including early life nutrition, dietary diversity, food environments and socioeconomic factors [7]. In low-and middle-income countries like Vietnam with persistent undernutrition problems and rising overweight and obesity, undernutrition focused programs may unintentionally increase risks for obesity [7]. Hawkes et al. proposed ten double-duty actions regarding breastfeeding, growth monitoring, food fortification and supplements, cash and food transfers, school feeding programs, nutrition and agriculture programs, food system policies, and food environments to address all forms of malnutrition [7].

This study has some limitations as the model is based on secondary data, and as modeling studies are generally underpinned by several assumptions. We used best source available, yet due to data paucity in Vietnam, the model relied on data from China for transition probabilities between weight status for adults. We could not apply mortality risks by weight status among children due to lack of data, and for mortality risks among adults, we used data from East Asia. In addition, overweight and obesity were not separated and considered as one weight status category ‘overweight/obesity’ due to limited availability of data for children with obesity in Vietnam. It may have affected study findings by under- or over-estimation, which is commonly described in other modelling studies [16, 44–46]. However, we performed a number of sensitivity and scenario analyses to illustrate how the results change if certain model inputs are altered. For instance, we conducted scenario analyses by altering transition probabilities to underweight or overweight/obesity by 10%, 20% and 50% to see improved outcomes by reducing the prevalence of underweight and overweight/obesity. The model would produce better estimations when country specific and more reliable data become available. As a life table modeling study, another limitation is that mortality rates and transition probabilities remain constant throughout the model time horizon, which has been also indicated in previous modeling study [44]. Lastly, our estimations were limited to population-level data and did not provide disaggregated outcomes by socioeconomic factors or health conditions due to data unavailability. Future research can estimate study outcomes by socioeconomic groups or other health conditions to have a broader understanding of impact of being underweight or overweight/obese with an equity perspective.

Despite the limitations, this study can provide useful information for policy makers to understand the magnitude of lifetime impact of child underweight and overweight/obesity. Through several scenario analyses, the model estimated possible health benefits from tackling underweight and overweight/obesity and burden from the rise in overweight/obesity. The government can utilize the data for their priority setting, resource allocation and program development. Improving child health and nutrition will contribute to achieving the National Nutrition Strategy in Vietnam and global Sustainable Development Goals, in particular ‘Goal 2: Zero Hunger’ and ‘Goal 3: Good Health and Well-being.’ Another strength of this study is that the model captured both underweight and overweight/obesity. The model structure is transferable to other low-and middle-income countries where both underweight and overweight/obesity risk child development. Other countries can replicate the model and estimate their own situation by changing input parameters, including prevalence of underweight and overweight/obesity and mortality rates. It would be a useful way to quantify the burden of malnutrition at the population level within limited resources. The model structure in Excel is available in Additional file 3 for further use. The model can be made freely available as an online platform tool so researchers from low-and middle-income countries can have access to the model and adapt to their local contexts and available data.

## Conclusions

This study quantified the lifetime impact of being underweight or overweight/obesity during childhood in Vietnam. Extensive scenario analyses showed that the reduction in underweight and overweight/obesity could result in fewer deaths, more years of life lived and more QALYs gained. Considering rapid changes with economic development, urbanization and nutrition transitions in Vietnam, tackling childhood underweight and overweight/obesity will be more crucial in improving health and nutrition. National policies such as the National Nutrition Strategy 2021–2030 for Vietnam and interventions in accordance with Sustainable Development Goals to address underweight and overweight/obesity are required for better life for every child and healthy society in Vietnam.

## Supplementary Information

**Additional file 1: Table 1.** Model inputs.

**Additional file 2:Supplementary Figure 1.** Differences in study outcomes ((**A**) deaths, (**B**) years of life lived, (**C**) QALYs) between various scenarios and base-case results.

**Additional file 3:** The model structure in Excel.

## Data Availability

The model structure in Excel is available in Additional file 3.

## Abbreviations

CI: Confidence interval
QALY: Quality-adjusted life years
