# Supplementary material for: Lifetime impact of being underweight or overweight/obese during childhood in Vietnam
Source: BMC Public Health. 2022 Apr 4;22:645. doi: 10.1186/s12889-022-13061-8 (PMC8981956; doi:10.1186/s12889-022-13061-8)
Supplement: Supplementary file 1 — Additional file 1: Table 1. Model inputs. [file 12889_2022_13061_MOESM1_ESM.docx]

**Additional file 1**

**Lifetime impact of being underweight or overweight during childhood in Vietnam**

Yeji Baek^1^, Alice J. Owen^1^, Jane Fisher^1^, Thach Tran^1,2^, Zanfina Ademi^1^

^1^ School of Public Health and Preventive Medicine, Monash University, Melbourne, Victoria, Australia

^2^ Research and Training Centre for Community Development, Hanoi, Vietnam

**Table 1. Model inputs**

|  | Men | | | Women | | | Distribution | Source |
| --- | --- | --- | --- | --- | --- | --- | --- | --- |
| Age | Underweight | Healthy weight | Overweight/  obesity | Underweight | Healthy weight | Overweight/  obesity |  |  |
| 5 | 12.3% | 66.9% | 20.8% | 12.9% | 76.3% | 10.9% | Uniform | 5-19 years: [1]  20+ years: [2] |
| 6 | 13.1% | 66.3% | 20.6% | 12.7% | 76.2% | 11.0% |  |  |
| 7 | 14.1% | 64.8% | 21.1% | 14.2% | 74.6% | 11.2% |  |  |
| 8 | 15.2% | 63.2% | 21.7% | 16.4% | 72.4% | 11.2% |  |  |
| 9 | 16.2% | 62.1% | 21.7% | 18.5% | 70.5% | 11.0% |  |  |
| 10 | 17.0% | 62.2% | 20.8% | 19.4% | 70.1% | 10.5% |  |  |
| 11 | 17.6% | 63.6% | 18.8% | 18.9% | 71.4% | 9.8% |  |  |
| 12 | 17.7% | 65.9% | 16.4% | 17.1% | 73.9% | 9.0% |  |  |
| 13 | 17.6% | 68.5% | 13.9% | 14.8% | 77.0% | 8.2% |  |  |
| 14 | 17.1% | 71.3% | 11.6% | 12.2% | 80.3% | 7.5% |  |  |
| 15 | 16.3% | 73.9% | 9.7% | 9.9% | 83.2% | 6.9% |  |  |
| 16 | 15.4% | 76.3% | 8.3% | 8.0% | 85.5% | 6.5% |  |  |
| 17 | 14.3% | 78.4% | 7.3% | 6.8% | 86.8% | 6.4% |  |  |
| 18 | 13.2% | 79.9% | 6.9% | 6.3% | 87.1% | 6.5% |  |  |
| 19 | 14.5% | 76.5% | 8.9% | 8.2% | 85.1% | 6.8% |  |  |
| 20+ | 16.8% | 66.7% | 16.4% | 17.9% | 60.8% | 21.3% |  |  |

1. **Prevalence of underweight, healthy weight and overweight/obesity**
2. **Transition probabilities between health states**

|  | Underweight (UW) | | | Healthy weight (HW) | | | Overweight/obesity (OWB) | | | Distribution | Source |
| --- | --- | --- | --- | --- | --- | --- | --- | --- | --- | --- | --- |
| Age | Stay UW | To HW | To OWB | Stay HW | To UW | To OWB | Stay OWB | To HW | To UW |  |  |
| Men | | | | | | | | | | Uniform | 5-22 years: [3]  23+ years: [4] |
| 5 to 8 | 0.429 | 0.556 | 0.016 | 0.817 | 0.113 | 0.070 | 0.727 | 0.273 | 0.000 |  |  |
| 9 to 12 | 0.609 | 0.381 | 0.009 | 0.860 | 0.085 | 0.055 | 0.713 | 0.273 | 0.014 |  |  |
| 13 to 15 | 0.520 | 0.480 | 0.000 | 0.877 | 0.091 | 0.031 | 0.583 | 0.417 | 0.000 |  |  |
| 16 to 22 | 0.420 | 0.568 | 0.012 | 0.819 | 0.107 | 0.074 | 0.647 | 0.353 | 0.000 |  |  |
| 23 to 30 | 0.400 | 0.568 | 0.032 | 0.856 | 0.042 | 0.102 | 0.777 | 0.217 | 0.006 |  |  |
| 31 to 40 | 0.404 | 0.590 | 0.006 | 0.827 | 0.023 | 0.150 | 0.837 | 0.163 | 0.000 |  |  |
| 41 to 50 | 0.459 | 0.525 | 0.016 | 0.830 | 0.020 | 0.150 | 0.812 | 0.186 | 0.003 |  |  |
| 51 to 60 | 0.557 | 0.434 | 0.008 | 0.824 | 0.031 | 0.145 | 0.810 | 0.188 | 0.002 |  |  |
| 61 to 75 | 0.548 | 0.433 | 0.019 | 0.817 | 0.049 | 0.134 | 0.767 | 0.231 | 0.002 |  |  |
| Women | | | | | | | | | |  |  |
| 5 to 8 | 0.513 | 0.475 | 0.013 | 0.876 | 0.080 | 0.045 | 0.596 | 0.404 | 0.000 |  |  |
| 9 to 12 | 0.548 | 0.446 | 0.005 | 0.867 | 0.083 | 0.049 | 0.551 | 0.449 | 0.000 |  |  |
| 13 to 15 | 0.404 | 0.596 | 0.000 | 0.957 | 0.024 | 0.019 | 0.541 | 0.459 | 0.000 |  |  |
| 16 to 22 | 0.629 | 0.371 | 0.000 | 0.766 | 0.202 | 0.032 | 0.333 | 0.667 | 0.000 |  |  |
| 23 to 30 | 0.377 | 0.619 | 0.004 | 0.852 | 0.064 | 0.084 | 0.576 | 0.424 | 0.000 |  |  |
| 31 to 40 | 0.443 | 0.557 | 0.000 | 0.843 | 0.027 | 0.130 | 0.790 | 0.208 | 0.002 |  |  |
| 41 to 50 | 0.452 | 0.540 | 0.008 | 0.812 | 0.024 | 0.164 | 0.833 | 0.167 | 0.000 |  |  |
| 51 to 60 | 0.541 | 0.439 | 0.020 | 0.812 | 0.033 | 0.154 | 0.782 | 0.215 | 0.003 |  |  |
| 61 to 75 | 0.578 | 0.410 | 0.012 | 0.780 | 0.052 | 0.168 | 0.793 | 0.204 | 0.003 |  |  |

UW, underweight; HW, healthy weight; OWB, overweight/obesity

1. **All-cause mortality**

| Age | Men | Women | Distribution | Source |
| --- | --- | --- | --- | --- |
| 5 to 9 | 0.0003 | 0.0001 | Lognormal | [5] |
| 10 to 14 | 0.0004 | 0.0002 |  |  |
| 15 to 19 | 0.0011 | 0.0004 |  |  |
| 20 to 24 | 0.0015 | 0.0004 |  |  |
| 25 to 29 | 0.0015 | 0.0005 |  |  |
| 30 to 34 | 0.0017 | 0.0006 |  |  |
| 35 to 39 | 0.0023 | 0.0009 |  |  |
| 40 to 44 | 0.0036 | 0.0015 |  |  |
| 45 to 49 | 0.0059 | 0.0023 |  |  |
| 50 to 54 | 0.0095 | 0.0036 |  |  |
| 55 to 59 | 0.0147 | 0.0055 |  |  |
| 60 to 64 | 0.0225 | 0.0089 |  |  |
| 65 to 69 | 0.0343 | 0.0147 |  |  |
| 70 to 74 | 0.0535 | 0.0258 |  |  |

1. **Mortality risk for 20 years old and over**

|  | Men | | | Women | | | Distribution | Source |
| --- | --- | --- | --- | --- | --- | --- | --- | --- |
|  | HR | 95% CI | | HR | 95% CI | |  |  |
| Underweight | 1.45 | 1.28 | 1.65 | 1.39 | 1.30 | 1.49 | Lognormal | [6] |
| Healthy weight | 1.00 | 0.96 | 1.04 | 1.00 | 0.98 | 1.02 |  |  |
| Overweight/obesity | 1.17 | 1.10 | 1.24 | 1.16 | 1.12 | 1.22 |  |  |

HR; hazard ratios, CI; confidence intervals.

1. **Quality of life, with utility scores**

|  | Men | | | Women | | | Distribution |  |
| --- | --- | --- | --- | --- | --- | --- | --- | --- |
| Age | Underweight | Healthy weight | Overweight/  obesity | Underweight | Healthy weight | Overweight/  obesity |  | Source |
| 5 to 17 | 1.000 | 1.000 | 1.000 | 1.000 | 1.000 | 1.000 | Beta | 5-17 years: authors’ assumption  18-75 years: [7] |
| 18 to 24 | 0.966 | 0.966 | 0.966 | 0.954 | 0.954 | 0.954 |  |  |
| 25 to 34 | 0.966 | 0.966 | 0.966 | 0.961 | 0.961 | 0.961 |  |  |
| 35 to 59 | 0.962 | 0.965 | 0.970 | 0.936 | 0.960 | 0.937 |  |  |
| 60 to 75 | 0.975 | 0.962 | 0.942 | 0.947 | 0.938 | 0.901 |  |  |

**References**

1. NCD Risk Factor Collaboration (NCD-RisC). Child & Adolescent Body-Mass Index [https://ncdrisc.org/data-downloads-adiposity-ado.html [Accessed 1 July 2021].
2. NCD Risk Factor Collaboration (NCD-RisC). National Adult Body-Mass Index [https://ncdrisc.org/data-downloads-adiposity.html [Accessed 1 July 2021].
3. Favara M, Crivello G, Penny M, Porter C, Revathi E, Sánchez A, et al. Cohort Profile Update: The Young Lives study. Int J Epidemiol. 2021; doi:10.1093/ije/dyab111.
4. Popkin BM, Du S, Zhai F, Zhang B. Cohort Profile: The China Health and Nutrition Survey--monitoring and understanding socio-economic and health change in China, 1989-2011. Int J Epidemiol. 2010;39(6):1435-40; doi:10.1093/ije/dyp322.
5. WHO Global Health Observatory data repository. Life table for Vietnam [https://apps.who.int/gho/data/view.main.61830?lang=en [Accessed 1 July 2021].
6. Global BMI Mortality Collaboration, Di Angelantonio E, Bhupathiraju Sh N, Wormser D, Gao P, Kaptoge S, et al. Body-mass index and all-cause mortality: individual-participant-data meta-analysis of 239 prospective studies in four continents. Lancet. 2016;388(10046):776-86; doi:10.1016/s0140-6736(16)30175-1.
7. Mai VQ, Giang KB, Minh HV, Lindholm L, Sun S, Sahlen KG. Reference data among general population and known-groups validity among hypertensive population of the EQ-5D-5L in Vietnam. Qual Life Res. 2021; doi:10.1007/s11136-021-02959-2.
