# Supplementary material for: Lifetime impact of being underweight or overweight/obese during childhood in Vietnam
Source: BMC Public Health. 2022 Apr 4;22:645. doi: 10.1186/s12889-022-13061-8 (PMC8981956; doi:10.1186/s12889-022-13061-8)
Supplement: Supplementary file 2 — Additional file 2:Supplementary Figure 1. Differences in study outcomes ((A) deaths, (B) years of life lived, (C) QALYs) between various scenarios and base-case results. [file 12889_2022_13061_MOESM2_ESM.docx]

**Additional file 2**

**Lifetime impact of being underweight or overweight during childhood in Vietnam**

Yeji Baek^1^, Alice J. Owen^1^, Jane Fisher^1^, Thach Tran^1,2^, Zanfina Ademi^1^

^1^ School of Public Health and Preventive Medicine, Monash University, Melbourne, Victoria, Australia

^2^ Research and Training Centre for Community Development, Hanoi, Vietnam

**Supplementary Figure**

(A)


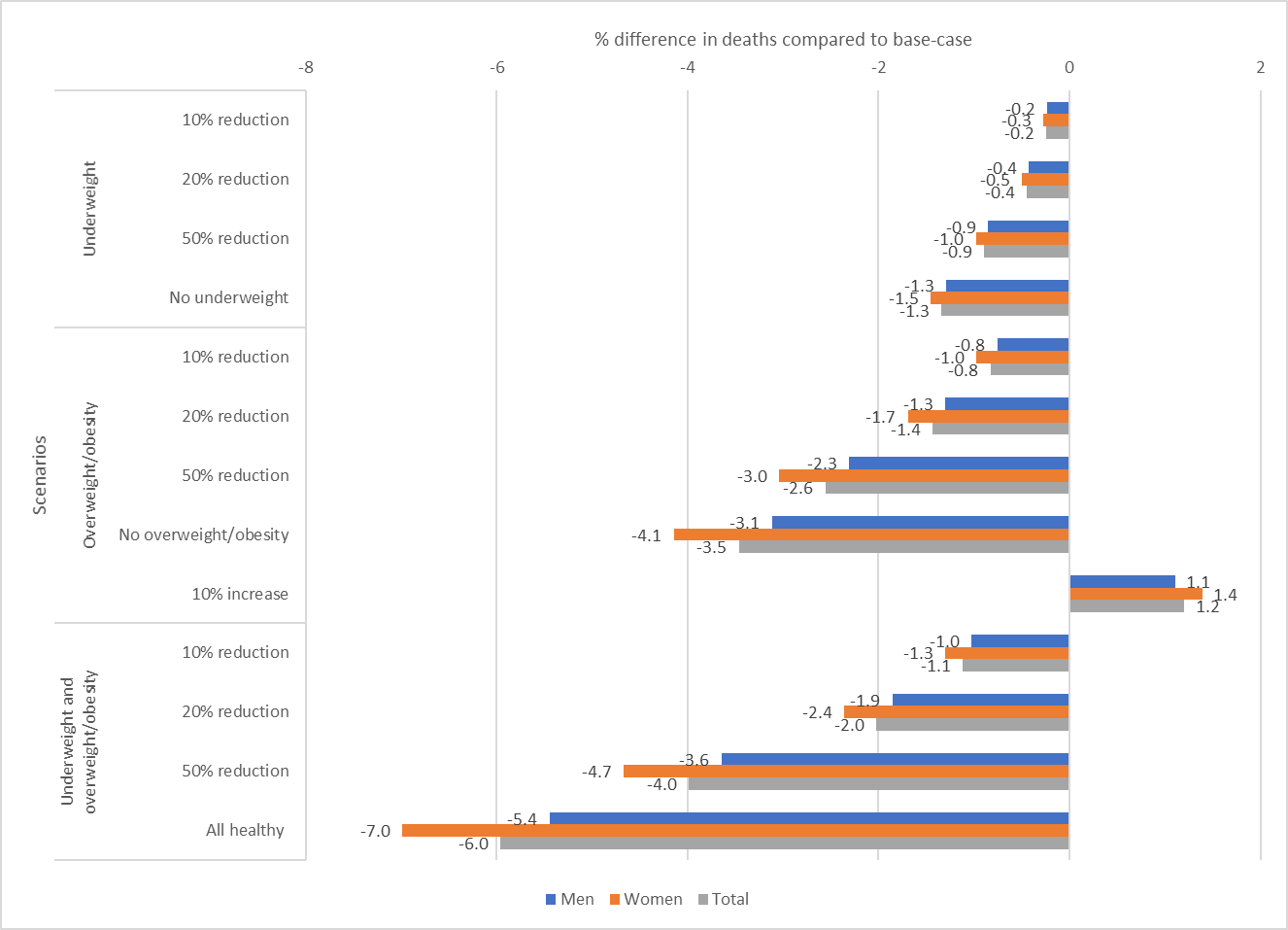


(B)


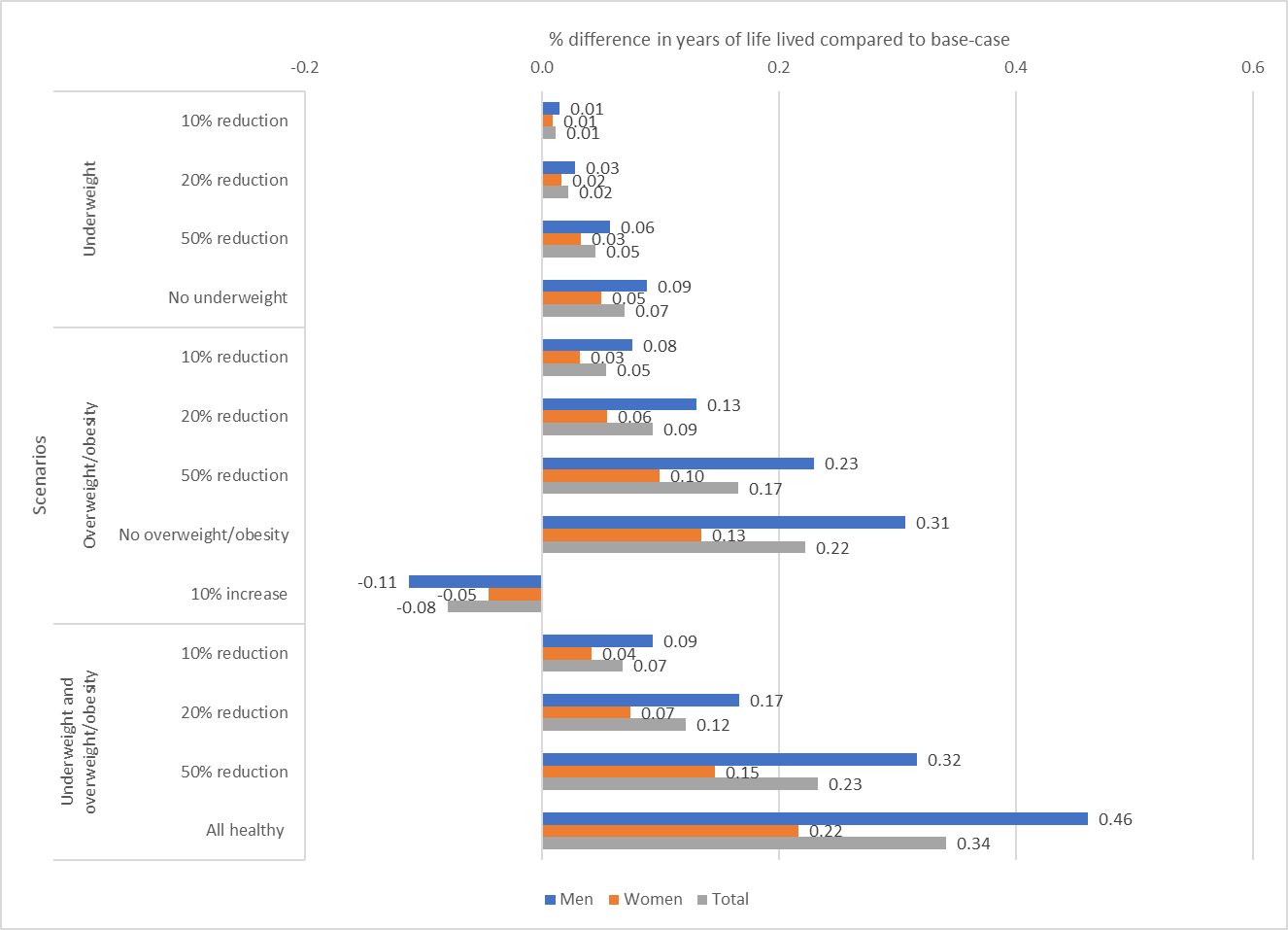


(C)
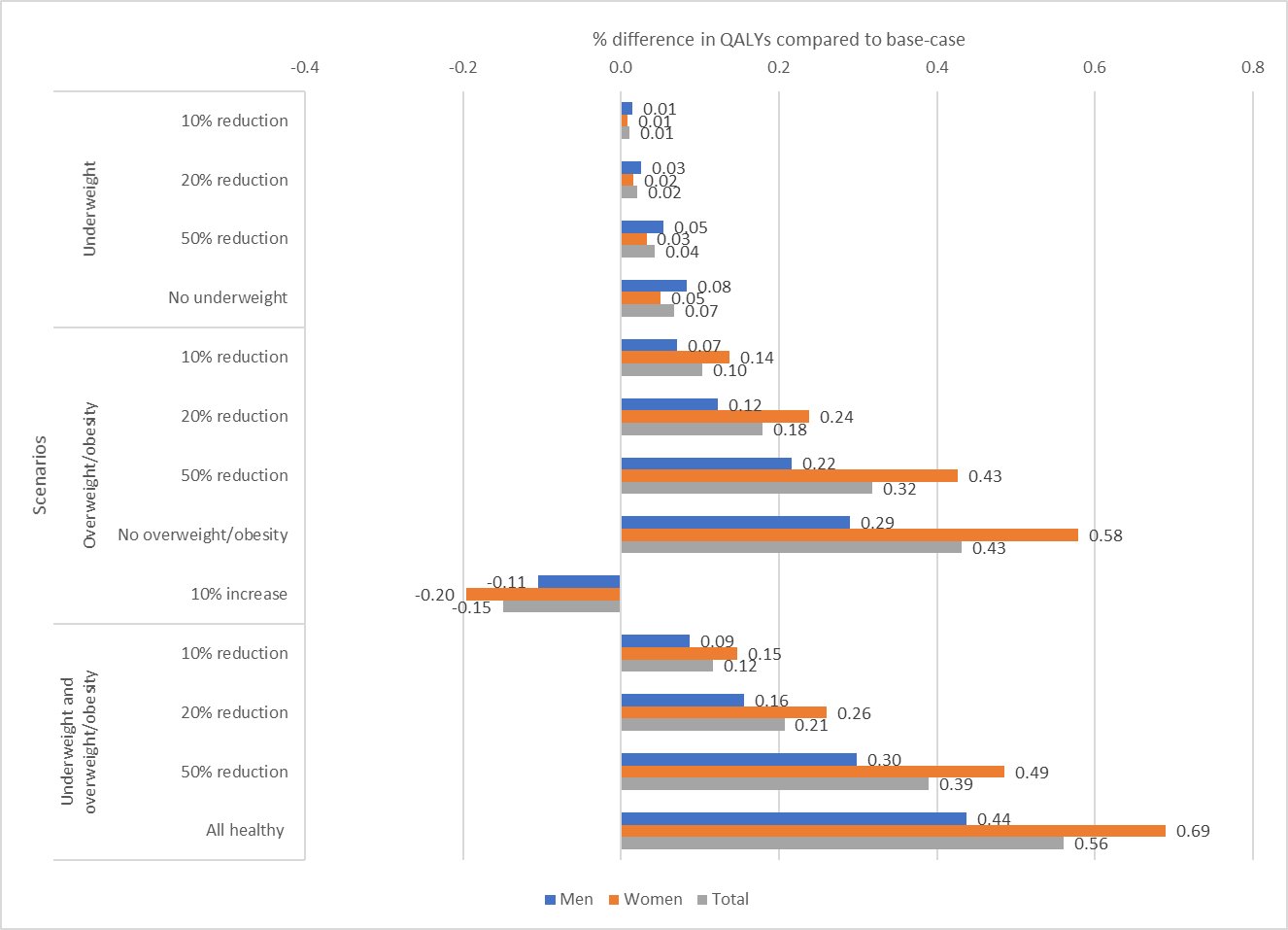


**Supplementary Figure 1. Differences in study outcomes ((A) deaths, (B) years of life lived, (C) QALYs) between various scenarios and base-case results**
